# Identifying key targets for interventions to improve psychological wellbeing: replicable results from four UK cohorts

**DOI:** 10.1017/S0033291718003288

**Published:** 2018-11-15

**Authors:** J. Stochl, E. Soneson, A.P. Wagner, G.M. Khandaker, I. Goodyer, P.B. Jones

**Affiliations:** 1 Department of Psychiatry, University of Cambridge, Cambridge Biomedical Campus, Herschel Smith Building for Brain & Mind Sciences, Cambridge, CB2 0SZ, Cambridge, UK; 2 National Institute for Health Research (NIHR) Collaboration for Leadership in Applied Health Research and Care East of England (CLAHRC), Cambridge, UK; 3 Department of Kinanthropology, Charles University, Prague, Czech Republic; 4 Norwich Medical School, University of East Anglia, Norwich, UK

**Keywords:** Intervention targets, mental health, network analysis, psychological wellbeing

## Abstract

**Background:**

An increasing importance is being placed on mental health and wellbeing at individual and population levels. While there are several interventions that have been proposed to improve wellbeing, more evidence is needed to understand which aspects of wellbeing are most influential. This study aimed to identify key items that signal improvement of mental health and wellbeing.

**Methods:**

Using network analysis, we identified the most central items in the graph network estimated from the well-established Warwick-Edinburgh Mental Well-being Scale (WEMWBS). Results were compared across four major UK cohorts comprising a total of 47,578 individuals: the Neuroscience in Psychiatry Network, the Scottish Schools Adolescent Lifestyle and Substance Use Survey, the Northern Ireland Health Survey, and the National Child Development Study.

**Results:**

Regardless of gender, the three items most central in the network were related to positive self-perception and mood: ‘I have been feeling good about myself’; ‘I have been feeling confident’; and ‘I have been feeling cheerful’. Results were consistent across all four cohorts.

**Conclusions:**

Positive self-perception and positive mood are central to psychological wellbeing. Psychotherapeutic and public mental health interventions might best promote psychological wellbeing by prioritising the improvement of self-esteem, self-confidence and cheerfulness. However, empirical testing of interventions using these key targets is needed.

## Introduction

Mental health and wellbeing are becoming increasingly prominent in national and international health policy (World Health Organization, 2002, 2004; Department of Health and Social Care, 2011; Mehta *et al*., 2015). At a societal level, they represent important resources closely linked to social, human and economic capital (Friedli and Parsonage, 2007; Knapp *et al*., 2011), and are associated with lower levels of inequality, less community violence and higher life expectancy (Friedli and World Health Organization, 2009). For individuals, mental health and wellbeing are closely connected to normal functioning and quality of life and are instrumental in creating and maintaining good relationships (World Health Organization, 2004; Jané-Llopis *et al*., 2005). Clinically, the growing evidence for the existence of a ‘continuum’ of psychopathology (also referred to as ‘common mental distress’ or the ‘general psychopathology factor’) (Caspi *et al*., 2014; Stochl *et al*., 2015) suggests that improving mental health and wellbeing may also help to prevent the development of mental disorders.

Several approaches have been suggested for improving mental health and wellbeing, including psychological therapies (Fava *et al*., 1998; Slade, 2010; Galante *et al*., 2017), school and workplace interventions (Jané-Llopis and Barry, 2005; Jané-Llopis *et al*., 2005; Knapp *et al*., 2011; Weare and Nind, 2011), improvement of housing and nutrition, reduction of substance misuse and prevention of violence (World Health Organization, 2004; Jané-Llopis *et al*., 2005). Despite their promise, however, many of these approaches have been criticised for their lack of supporting empirical evidence (Mehta *et al*., 2015). Indeed, current methods used to inform intervention targets are mainly limited to theoretical models (e.g. Ryff's model of wellbeing; general stress theory), literature reviews and qualitative methods (e.g. interviews with experts and service users), and do not consider any type of quantitative method.

Psychological network analysis is an innovative statistical approach that can complement theoretical knowledge and clinical expertise by providing quantitative evidence for the identification of intervention targets. Essentially, it examines relationships between different items on clinical questionnaires, and determines which items are most ‘central’ to the condition of interest due to having strong relationships with other items. Central items may then serve as indicators for clinical intervention targets (Fried *et al*., 2017), as their improvement is most likely to destabilise harmful network structures and prevent exacerbation of other items (Smith *et al*., 2018). Network analysis has been used to suggest potential intervention targets for depression (van Borkulo *et al*., 2015), post-traumatic stress disorder (Fried *et al*. 2018) and eating disorders (Smith *et al*., 2018). Furthermore, it aligns with the clinical characterisation of psychopathology as a system of causal relationships between symptoms, where some symptoms are more influential than others (van Borkulo *et al*., 2015).

To make valid inferences in network analysis, comprehensive tools to measure mental health and wellbeing, such as the well-established Warwick-Edinburgh Mental Well-being Scale (WEMWBS), are crucial. In this study, we have used psychological network analysis to identify items central to the WEMWBS, which we present as potentially optimal targets for interventions aiming to improve mental health and wellbeing.

## Methods

### Participants

This study sample comprises 47 578 participants from four different UK cohorts.

#### National Child Development Study

The National Child Development Study (NCDS) (University of London, 2012) is a major longitudinal British cohort study initiated in 1958. As such, this sample is homogeneous for age. At age 53, 8643 NCDS participants (51.8% women) completed the WEMWBS as part of a set of self-report questionnaires. Full details on sampling design and data collection can be found at https://tinyurl.com/y7q2m66z.

#### Northern Ireland Health Survey

The Northern Ireland Health Survey (NIHS) (Department of Health Northern Ireland, 2016) covers a range of health topics important to the lives of people in Northern Ireland. The survey has been annually conducted since 2010. Respondents are sampled from those aged 16+ living in private households. The 2010–2011 survey collected wellbeing data from 4161 individuals of which 3873 (58.8% women) had complete WEMWBS data. Details about the data collection methodology can be found at https://tinyurl.com/ybfakdsm.

#### Neuroscience in Psychiatry Network

The Neuroscience in Psychiatry Network (NSPN) (Kiddle *et al*., 2018) cohort consists of 2403 participants, aged 14–25, recruited from Cambridgeshire, London and surrounding areas. The sample analysed here was recruited between November 2012 and July 2017. Study invites were sent through general practice surgeries and schools with the aim of recruiting 200 women and 200 men for each of five age strata (ages: 14–15; 16–17; 18–19; 20–21; 22–24). Complete WEMWBS data were available from 2337 individuals (53.8% women).

#### Scottish Schools Adolescent Lifestyle and Substance Use Survey

The Scottish Schools Adolescent Lifestyle and Substance Use Survey (SALSUS) (NHS National Services Scotland, 2013) survey was set up by the Scottish Government to monitor progress on reducing smoking and substance misuse. Information from the survey helps national planning and facilitates the monitoring of policy implementation. The WEMWBS data used in this study were collected in 2010 from 32 725 individuals (49.4%, women) from the second (age 12–14) and fourth (age 14–16) years of secondary school. Full details can be found at https://tinyurl.com/ya66mdq4.

### The Warwick–Edinburgh Mental Well-being Scale

The WEMWBS (Tennant *et al*., 2007) is a 14-item, self-report measure designed to assess a range of wellbeing concepts including affective-emotional aspects, cognitive-evaluative dimensions and psychological functioning in the general population. All items are worded positively and have five response categories (1 – none of the time; 2 – rarely; 3 – some of the time; 4 – often; 5 – all of the time). The wellbeing score is computed as sum of all items (range: 14–70), with higher scores representing better wellbeing. The WEMWBS was found to be a unidimensional measure and to have desirable psychometric properties (Tennant *et al*., 2007). The scale is well-regarded by service users and their carers, who tend to prefer it to other mental health and wellbeing measures (Crawford *et al*., 2011) for the way that it asks about positive aspects of mental health.

### Analysis

Psychological network analysis (Borsboom and Cramer, 2013) conceptualises behaviour as a complex interplay of psychological and other components. Recently, this methodology has become popular in psychometrics partly due to its ability to identify worthwhile items for intervention development in questionnaires and surveys. In typical network analysis applied to questionnaire data (Gaussian graphical models), nodes (representing questionnaire items) are interconnected via edges (representing partial correlations) (Costantini *et al*., 2015). The use of partial correlations ensures that bivariate relationships between nodes are not confounded by relationships to other variables in the network and provides unbiased computation of centrality indices. Networks in this paper utilise the ‘spring’ layout (Fruchterman and Reingold, 1991), where nodes are positioned on a plane so that distances between them relate to the size of their partial correlations.

Typically, the network in each cohort is estimated separately and sparsity (and thus improved interpretability) of such networks is achieved by the application of an adaptive graphical LASSO penalty (Friedman *et al*., 2008). However, recent developments allow for joint estimation of multiple networks using *fused graphical LASSO* (FGL) (Danaher *et al*., 2014). FGL extends traditional graphical LASSO by extending the penalty function to incorporate differences among corresponding edge-weights estimated across networks. This strategy neither masks nor inflates similarities across networks (Fried *et al*., 2018). In this study, the optimal value of this penalty was achieved by *k*-fold cross-validation. A detailed explanation of FGL and its use in psychological networks is given elsewhere (Danaher *et al*., 2014; Fried *et al*., 2018; Costantini *et al*., 2019). The similarity of networks was assessed by calculating the Spearman correlation of edge-weights between each pair of networks (Borsboom, 2017).

The relative importance of questionnaire items is subsequently evaluated using measures from graph theory, using typical centrality indices such as *strength*, *closeness* and *betweenness* (Newman, 2010). A *strong* central node (item) (Barrat *et al*., 2004) is one that can influence many other nodes (or be influenced by them) directly, without considering the mediating role of other nodes (Costantini *et al*., 2015). As such, *strength* is the crucial index for identification of items for developing the most effective interventions. Nodes with high *closeness* (defined as the inverse of the sum of distances of the focal node to all other nodes in the network) are those whose responses are likely to be quickly affected by changes in other nodes, either directly or indirectly. If nodes with high *betweenness* are removed from a network, then the distance among other nodes will generally increase (Costantini *et al*., 2015). As such, nodes with high *betweenness* speed up the flow of information in networks.

Lack of accuracy and network stability have been recognised as an important issue in psychological networks (Forbes *et al*., 2017; Epskamp *et al*., 2018). Thus, bootstrapping procedures have been developed for psychological networks to address this issue and prevent biased inferences about the importance of individual nodes (Epskamp *et al*., 2018). To evaluate accuracy and stability, we have followed recommendations made by Epskamp *et al*. (2018). They proposed the correlation stability (CS) coefficient to investigate the stability of the order of centrality indices after observing only portions of the data. Its computation is based on case dropping bootstrap methods. The CS coefficient can be interpreted as the maximum proportion of cases that can be dropped, such that with 95% probability, the correlation between the original centrality indices and the centrality of networks based on subsets is 0.7 or higher [this figure can be changed but is taken as a default based on a simulation study by Epskamp *et al*. (2018)]. This coefficient should not drop below 0.25 and should ideally be above 0.5 to justify robust interpretation of centrality indices.

Functions from the R (R Core Team, 2017) packages ‘qgraph’ (Epskamp *et al*., 2012), ‘EstimateGroupNetwork’ (Costantini and Epskamp, 2017) and ‘mgm’ (Haslbeck and Waldorp, 2016) were used to estimate the network graphs. Given that the WEMWBS items are ordinal, polychoric correlations are used in the input weight matrix. The resulting networks were plotted using the *spring* layout (Fruchterman and Reingold, 1991) where more related edges are plotted closer together. Bootstrapping of networks was accomplished using the R package ‘bootnet’ (Epskamp *et al*., 2018). To assess network differences (global network *strength*, edges) with respect to gender, permutation tests implemented in the package ‘NetworkComparisonTest’ (van Borkulo *et al*., 2016) were used with 5000 iterations. All *p* values were corrected for multiple testing (using Holm–Bonferroni correction), where applicable.

### Ethical approvals

Ethical approval was not required for the present secondary data analysis.

## Results

Table 1 shows the basic item descriptive statistics for each cohort.

**Table 1.** WEMWBS item labels, wording and item means (standard deviations) across samples

Item	Statement	Mean (standard deviation)			
		NCDS	NIHS	NSPN	SALSUS
i1	I have been feeling optimistic about the future	3.28 (0.87)	3.23 (1.06)	3.44 (0.98)	3.25 (1.08)
i2	I have been feeling useful	3.56 (0.80)	3.50 (0.99)	3.25 (0.92)	3.21 (0.97)
i3	I have been feeling relaxed	3.30 (0.81)	3.32 (0.96)	3.22 (0.94)	3.41 (0.98)
i4	I have been feeling interested in other people	3.54 (0.82)	3.56 (0.97)	3.56 (0.91)	3.42 (1.04)
i5	I have had energy to spare	2.81 (0.91)	2.85 (1.06)	2.94 (1.02)	3.48 (1.06)
i6	I have been dealing with problems well	3.59 (0.78)	3.59 (0.90)	3.35 (0.95)	3.46 (1.06)
i7	I have been thinking clearly	3.71 (0.75)	3.82 (0.90)	3.54 (0.94)	3.65 (1.00)
i8	I have been feeling good about myself	3.39 (0.88)	3.57 (0.96)	3.40 (1.00)	3.49 (1.08)
i9	I have been feeling close to other people	3.58 (0.84)	3.73 (0.93)	3.53 (0.99)	3.72 (1.02)
i10	I have been feeling confident	3.46 (0.88)	3.52 (0.97)	3.37 (1.02)	3.54 (1.06)
i11	I have been able to make up my own mind about things	3.96 (0.79)	4.01 (0.87)	3.63 (0.98)	4.04 (0.93)
i12	I have been feeling loved	3.91 (0.99)	4.04 (0.98)	3.77 (1.08)	3.93 (1.07)
i13	I have been interested in new things	3.60 (0.90)	3.51 (1.02)	3.68 (1.00)	3.73 (1.02)
i14	I have been feeling cheerful	3.58 (0.81)	3.63 (0.86)	3.57 (0.95)	3.79 (1.00)

**Fig. 1.**
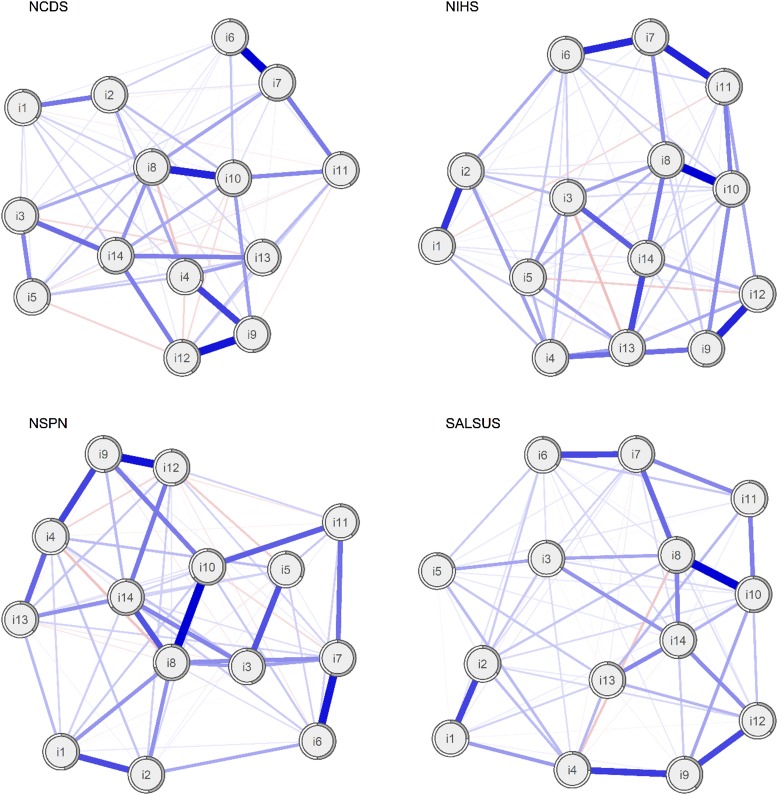
Networks of WEMWBS items in four general population samples. Nodes represent WEMWBS items and edges partial correlations with LASSO penalty. Distances between nodes and the thickness of edges relate to the size of their partial correlations. Grey doughnut charts surrounding each node show its explained variance.

Estimated networks are shown in Fig. 1. Visual comparison reveals similarities across them: for example, items 8 (*I have been feeling good about myself*) and 14 (*I have been feeling cheerful*) are always central. Item 10 (*I have been feeling confident*) seems to have a more prominent role in both the older (NCDS) and younger adult (NSPN) cohorts. Conversely, items such as 1 (*I have been feeling optimistic about the future*), 2 (*I have been feeling useful*) and 5 (*I have had energy to spare*) are generally on the periphery of the networks and less connected with other items. The formal comparison of networks (using a permutation test) revealed statistically significant differences in global network *strength* between NCDS and SALSUS (network strength NCDS = 6.75, network strength SALSUS = 6.23, *p* < 0.001) and also between NIHS and SALSUS (network strength NIHS = 6.54, network strength SALSUS = 6.23, *p* = 0.002). On average, around six edges between each pair of networks are statistically different. Information about significant differences in edge-weights is available from the authors upon request. We formally compare centrality indices later in this paper.

### Comparison of edge-weights and their accuracy

**Fig. 2.**
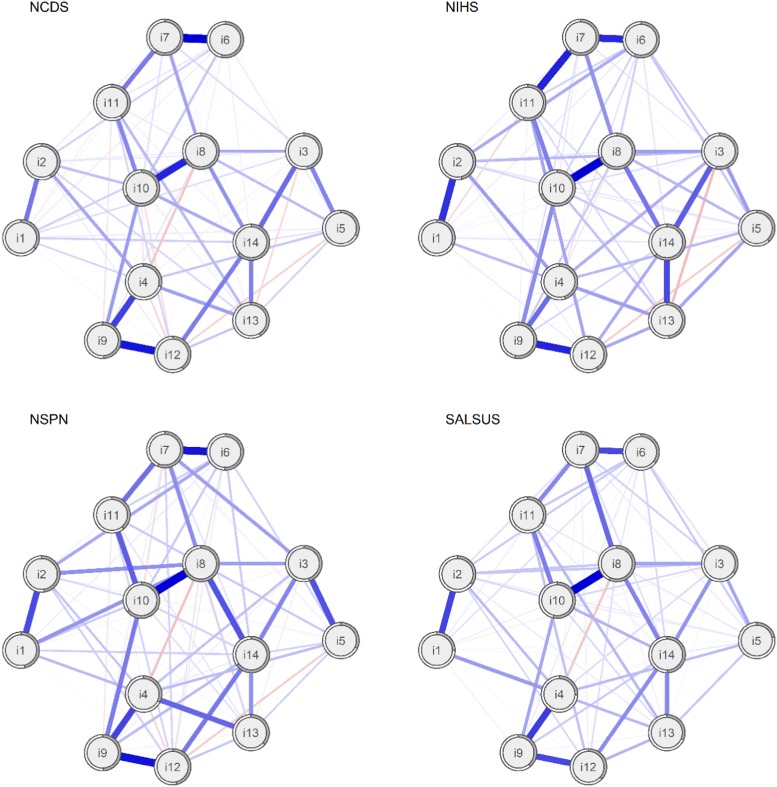
Networks of WEMWBS items in four general population samples using average spring layout. Nodes represent WEMWBS items and edges partial correlations with LASSO penalty. Distances among nodes and thickness of edges relate to size of their partial correlations. Grey doughnut charts surrounding each node show its explained variance.

To improve visual comparability of edges, we also estimated the average layout of these four networks and plotted all networks using this layout (see Fig. 2). The patterns of relationships among items are similar across samples. Items 8 and 10, which evaluate self-perception, are highly related. The same holds for items 4, 9 and 12, which assess relationships with other people, and items 6, 7 and 11, which deal with processing ideas and problems. It is less clear why items 1 (*I have been feeling optimistic about the future*) and 2 (*I have been feeling useful*) are related.

The visual similarity of networks was confirmed by investigating Spearman correlations of edge-weights for all pairs of networks, presented in online Supplementary Table S1. They ranged from 0.75 to 0.87, suggesting high similarity across networks.

Online Supplementary Fig. S1 depicts point estimates and bootstrap confidence intervals of the edge values for each network. In general, confidence intervals suggest that the accuracy of edges is satisfactory. As expected, the confidence intervals are smaller in larger samples.

### Centrality indices and their stability

**Fig. 3.**
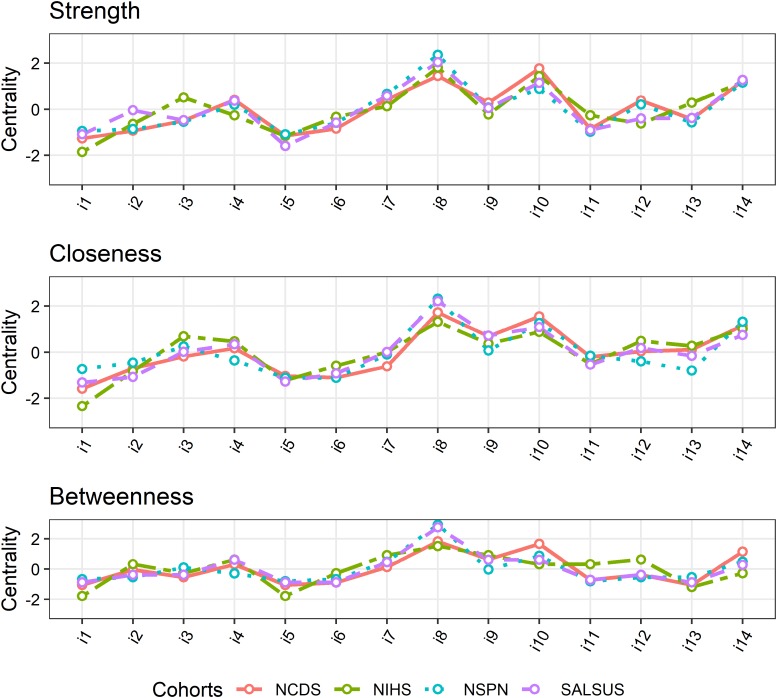
Centrality indices across cohorts.

Standardised centrality indices for each item, computed for each network, are shown in Fig. 3. The indices are remarkably similar across all networks. With respect to *strength* and *closeness*, the three most central items across all networks are items 8, 10 and 14. *Betweenness* of these three items is also highest in NCDS and NSPN. The top three *betweenness* items in NIHS are items 8, 7 and 9. In SALSUS, the top *betweenness* item is item 8 but next highest *betweenness* is indistinguishable for items 4, 9 and 10. These results suggest that the wellbeing intervention targets (as measured by *strength*) replicate well across cohorts. The same holds for *closeness*. Mediating items which speed up influence of changes in the network (*betweenness*) vary only slightly across cohorts.

Stability of the centrality indices was assessed using the case dropping bootstrap (Epskamp *et al*., 2018). The results from are plotted in online Supplementary Fig. S2, and corresponding CS coefficients are given in Table 2.

**Table 2.** Correlation stability coefficients

	Betweenness	Closeness	Strength
NCDS	0.67	>0.75	>0.75
NIHS	0.36	>0.75	>0.75
NSPN	0.67	>0.75	>0.75
SALSUS	>0.75	>0.75	>0.75

These results show that *closeness* and *strength* are very stable (even with only 25% of cases, the order of centrality indices has not considerably changed). *Betweenness* is slightly less stable, but apart from NIHS sample, its confidence intervals are still above the recommended cut-off of 0.5. Therefore, *betweenness* in NIHS should be interpreted with caution.

### Gender differences

Network structures, and thus wellbeing intervention targets, might be different for men and women. We have therefore tested for statistically significant gender differences in global network *strength* and edge-weights. Regardless of cohort, there were no statistically significant differences by gender in global network *strength* (*p* values: NCDS = 0.163; NIHS = 0.422; NSPN = 0.696; SALSUS = 0.474). No differences in edge-weights were found in the NIHS or NSPN cohorts. In NCDS, a significant difference between men and women was found for the edge between items 8 and 10 (0.33 for men, 0.45 for women, *p* = 0.035). This suggests the link between item 8 (*feeling good about oneself*) and item 10 (*feeling confident*) is stronger for middle-aged women than for middle-aged men. Even in the very large SALSUS cohort (*n* = 32 725), only six edges (out of 91) were significantly different between men and women [*p*(item 8, item 9) < 0.001; *p*(item 8, item 10)<0.001; *p*(item 2, item 10) < 0.001; *p*(item 7, item 10) < 0.001; *p*(item 8, item 14) < 0.001; *p*(item 9, item 14) = 0.017]. In this cohort, links between (1) item 8 (*feeling good about oneself*) and item 10 (*feeling confident*) and (2) item 9 (*feeling close to others*) and item 14 (*feeling cheerful*) were stronger for women than for men. Conversely, links between (1) item 2 (*feeling useful*) and item 10 (*feeling confident*), (2) item 7 (*thinking clearly*) and item 10 (*feeling confident*), (3) item 8 (*feeling good about oneself*) and item 9 (*feeling close to others*), and (4) item 8 (*feeling good about oneself*) and item 14 (*feeling cheerful*) were all stronger for men than for women. On the whole, the relatively small number of significantly different edges suggests that gender differences in these wellbeing networks are minimal.

## Discussion

This study aimed to identify the central aspects of psychological wellbeing, which may thus be considered as important intervention targets. Score improvements on these items should have the largest positive impact on other aspects of psychological wellbeing. To find these keystones, we used psychological network analysis to identify the most central items within graph networks created from a well-established psychological wellbeing measure (WEMWBS). The WEMWBS data were obtained from four major UK cohorts varying with respect to age [young people (SALSUS), adolescents and young adults (NSPN), general adult population (NIHS) and middle-aged adults (NCDS)] and location (England, Northern Ireland and Scotland).

Generally, results were consistent across cohorts. Edge-weights showed very similar patterns across cohorts and were accurate enough to make valid inferences about network architecture. This suggests high replicability of the network structure and high generalisability of findings across ages and geographical locations within the UK.

To highlight optimal targets that maximise intervention effectiveness, the most important items are those central to a network. The top three items, as measured by *strength* are items 8 (*I have been feeling good about myself*), 10 (*I have been feeling confident*) and 14 (*I have been feeling cheerful*). This suggests that positive self-perception and cheerfulness may play the most important role in influencing other aspects of psychological wellbeing. Due to the undirected character of the network, it is not surprising that these items demonstrate the highest levels of *closeness*, indicating that they are easily influenced by other network nodes. The least influential items vary slightly across samples, but often include items 1 (*I have been feeling optimistic about the future*), 5 (*I have had energy to spare*), 6 (*I have been dealing with problems well*) and 11 (*I have been able to make up my own mind about things*). This suggests that improving upon processing problems, energy and future expectations may have the smallest effect on other aspects of wellbeing.

These inferences seem to be robust given the high stability of centrality indices. Apart from *betweenness* in the NIHS cohort (which has questionable interpretability due to poor stability), all other CS coefficients were above the recommended criteria of 0.50 (Epskamp *et al*., 2018).

Gender differences in network architecture (global *strength*, size of edges) were also assessed to determine if intervention targets might differ for men and women. Omnibus tests of global network *strength* suggested no gender differences in any sample. Given there was only a total of seven edge differences by gender across all four cohorts, our results suggest that intervention targets are unlikely to differ by gender.

### Strengths and limitations

A key strength of this study is that it utilises a number of cohorts, addressing the considerable concern about the replicability crisis in network literature (Forbes *et al*., 2017). In addition, the considered cohorts are large and cover a wide range of age and geographical locations, supporting the generalisability of findings.

A substantial limitation is the use of cross-sectional data, which constrains network analysis to undirected networks. Using undirected networks in turn limits inferences about the direction of influence. Although presented network edges can be interpreted as putative causal paths, it is equally likely that influence flows from A to B as from B to A (other scenarios are also possible including mediation by another node C). Indeed, it seems plausible that feeling good about yourself (item 8), being confident (item 10) and feeling cheerful (item 14) might be the consequence rather than cause of other aspects of wellbeing considered here (e.g. feeling relaxed, loved by others or thinking positively about the future). An intervention affecting only the end-points of a causality chain, as in this scenario, is likely to have only limited, if any, the impact on mental wellbeing (Fried *et al*., 2018). Experimental studies that intervene directly on the central symptoms are therefore needed to test whether this would indeed affect other symptoms in an expected way (Fried and Cramer, 2017).

Additionally, as clearly described in Fried *et al*. (2018), there are at least two other reasons why using central items as intervention targets should be considered with caution. First, feedback loops, which are difficult to detect in undirected networks, can make central items the most resilient to change. Second, peripheral items should not automatically be regarded as clinically unimportant; their importance should be also considered based on substantive clinical arguments. However, despite all these limitations, Fried *et al*. (2018, p. 11) conclude, ‘If we had to put our money on selecting a clinical feature as an intervention target in the absence of all other clinical information, […] choosing the most central node might be a viable heuristic.’

### Implications for practice

Our findings have implications for the design of national mental health and wellbeing strategies for all ages. Positive self-perception and confidence in children and young people could be improved effectively at schools (e.g. bullying prevention programmes) or at home (e.g. positive parenting programmes), and in adults at the workplace (e.g. through regular training and supervision; fostering positive and supporting working environments). Indeed, the UK government expects schools and employers to play active roles in promoting population mental health and wellbeing (Department of Health and Social Care, 2011). Furthermore, although our findings are based on general population samples, they may be useful for providing care for people seeking treatment for mental disorders. Since evidence suggests that psychological wellbeing and mental ill health exist on a continuum (Caspi *et al*., 2014; Stochl *et al*., 2015; Böhnke and Croudace, 2016; St Clair *et al*., 2017), it is likely that improving wellbeing in mentally unwell individuals may also lead to improvements in their clinical symptoms. Finally, our analysis may also have implications for the development and trialling of psychological therapies as they indicate that interventions that focus on improving self-esteem and confidence may be more effective in increasing overall wellbeing than those that do not focus on these qualities.

## Conclusions

In conclusion, our study shows that the most worthwhile intervention targets for improvement of psychological wellbeing are aspects related to positive self-perception and positive mood. Regardless of gender, their improvement is likely to have a positive impact on the remaining aspects of psychological wellbeing, either directly or indirectly.

## Supplementary material

For supplementary material accompanying this paper visit https://doi.org/10.1017/S0033291718003288. click here to view supplementary material

## References

[ref1] Barrat A, Barthelemy M, Pastor-Satorras R and Vespignani A (2004) The architecture of complex weighted networks. Proceedings of the National Academy of Sciences of the USA 101, 3747–3752. 15007165 10.1073/pnas.0400087101 PMC374315

[ref2] Böhnke JR and Croudace TJ (2016) Calibrating well-being, quality of life and common mental disorder items: psychometric epidemiology in public mental health research. British Journal of Psychiatry 209, 162–168. 26635327 10.1192/bjp.bp.115.165530 PMC4967770

[ref3] Borsboom D (2017) A network theory of mental disorders. World Psychiatry 16, 5–13. 28127906 10.1002/wps.20375 PMC5269502

[ref4] Borsboom D and Cramer AO (2013) Network analysis: an integrative approach to the structure of psychopathology. Annual Review of Clinical Psychology 9, 91–121. 10.1146/annurev-clinpsy-050212-185608 23537483

[ref5] Caspi A, Houts RM, Belsky DW, Goldman-Mellor SJ, Harrington H, Israel S, Meier MH, Ramrakha S, Shalev I, Poulton R and Moffitt TE (2014) The p factor: one general psychopathology factor in the structure of psychiatric disorders? Clinical Psychological Science 2, 119–137. 25360393 10.1177/2167702613497473 PMC4209412

[ref6] Costantini G and Epskamp S (2017) Estimate Group Network: Perform the Joint Graphical Lasso and Selects Tuning Parameters. R package version 0.1.2.

[ref7] Costantini G, Epskamp S, Borsboom D, Perugini M, Mõttus R, Waldorp LJ and Cramer AO (2015) State of the aRt personality research: a tutorial on network analysis of personality data in R. Journal of Research in Personality 54, 13–29.

[ref8] Costantini G, Richetin J, Preti E, Casini E, Epskamp S and Perugini M (2019) Stability and variability of personality networks. A tutorial on recent developments in network psychometrics. Personality and Individual Differences 136, 68–78.

[ref9] Crawford MJ, Robotham D, Thana L, Patterson S, Weaver T, Barber R, Wykes T and Rose D (2011) Selecting outcome measures in mental health: the views of service users. Journal of Mental Health 20, 336–346. 21770782 10.3109/09638237.2011.577114

[ref10] Danaher P, Wang P and Witten DM (2014) The joint graphical lasso for inverse covariance estimation across multiple classes. Journal of the Royal Statistical Society: Series B *(*Statistical Methodology*)* 76, 373–397. 24817823 10.1111/rssb.12033 PMC4012833

[ref11] Department of Health and Social Care (2011) No Health Without Mental Health: A Cross-Government Mental Health Outcomes Strategy for People of all Ages. London, UK: Stationery Office.

[ref12] Department of Health Northern Ireland (2016) Northern Ireland Health Survey, 2010–2011. UK Data Service.

[ref13] Epskamp S, Cramer AOJ, Waldorp LJ, Schmittmann VD and Borsboom D (2012) Qgraph: network visualizations of relationships in psychometric data. Journal of Statistical Software 48, 1–18.

[ref14] Epskamp S, Borsboom D and Fried EI (2018) Estimating psychological networks and their accuracy: a tutorial paper. Behavior Research Methods 50, 195–212. 28342071 10.3758/s13428-017-0862-1 PMC5809547

[ref15] Fava GA, Rafanelli C, Cazzaro M, Conti S and Grandi S (1998) Well-being therapy. A novel psychotherapeutic approach for residual symptoms of affective disorders. Psychological Medicine 28, 475–480. 9572104 10.1017/s0033291797006363

[ref16] Forbes MK, Wright AG, Markon KE and Krueger RF (2018) Evidence that psychopathology symptom networks have limited replicability. Journal of Abnormal Psychology 126, 969. 10.1037/abn0000276 PMC5749927 29106281

[ref17] Fried EI and Cramer AO (2017) Moving forward: challenges and directions for psychopathological network theory and methodology. Perspectives on Psychological Science 12, 999–1020. 28873325 10.1177/1745691617705892

[ref18] Fried EI, van Borkulo CD, Cramer AOJ, Boschloo L, Schoevers RA and Borsboom D (2017) Mental disorders as networks of problems: a review of recent insights. Social Psychiatry and Psychiatric Epidemiology 52, 1–10. 27921134 10.1007/s00127-016-1319-z PMC5226976

[ref19] Fried EI, Eidhof MB, Palic S, Costantini G, Huisman-van Dijk HM, Bockting CL, Engelhard I, Armour C, Nielsen AB and Karstoft K-I (2018) Replicability and generalizability of posttraumatic stress disorder (PTSD) networks: a cross-cultural multisite study of PTSD symptoms in four trauma patient samples. Clinical Psychological Science 6, 335–351. 29881651 10.1177/2167702617745092 PMC5974702

[ref20] Friedli L and Parsonage M (2007) Building an economic case for mental health promotion: part I. Journal of Public Mental Health 6, 14–23.

[ref21] Friedli L and World Health Organization (2009) Mental health, resilience and inequalities.

[ref22] Friedman J, Hastie T and Tibshirani R (2008) Sparse inverse covariance estimation with the graphical lasso. Biostatistics (Oxford, England) 9, 432–441. 10.1093/biostatistics/kxm045 PMC3019769 18079126

[ref23] Fruchterman TMJ and Reingold EM (1991) Graph drawing by force-directed placement. Software: Practice and Experience 21, 1129–1164.

[ref24] Galante J, Dufour G, Vainre M, Wagner AP, Stochl J, Benton A, Lathia N, Howarth E and Jones PB (2017) A mindfulness-based intervention to increase resilience to stress in university students (the Mindful Student Study): a pragmatic randomised controlled trial. Lancet Public Health 3, e72–e81. 29422189 10.1016/S2468-2667(17)30231-1 PMC5813792

[ref25] Haslbeck JM and Waldorp LJ (2016) Mgm: structure estimation for time-varying mixed graphical models in high-dimensional data. Journal of Statistical Software.

[ref26] Jané-Llopis E and Barry MM (2005) What makes mental health promotion effective? Promotion & Education 12, 47–54. 10.1177/10253823050120020108 15966253

[ref27] Jané-Llopis E, Barry M, Hosman C and Patel V (2005) Mental health promotion works: a review. Promotion & Education 12, 9–25. 10.1177/10253823050120020103x 15966248

[ref28] Kiddle B, Inkster B, Prabhu G, Moutoussis M, Whitaker KJ, Bullmore ET, Dolan RJ, Fonagy P, Goodyer IM and Jones PB (2018) Cohort profile: the NSPN 2400 Cohort: a developmental sample supporting the Wellcome Trust NeuroScience in Psychiatry Network. International Journal of Epidemiology 47, 18–19g. 29177462 10.1093/ije/dyx117 PMC5837633

[ref29] Knapp M, McDaid D and Parsonage M (2011) Mental health promotion and mental illness prevention: the economic case.

[ref30] Mehta N, Croudace T and Davies SC (2015) Public mental health: evidenced-based priorities. Lancet 385, 1472–1475. 25217115 10.1016/S0140-6736(14)61400-8

[ref31] Newman M (2010) Networks: An Introduction. New York, NY: Oxford University Press.

[ref32] NHS National Services Scotland (2013) Scottish Schools Adolescent Lifestyle and Substance Use Survey, 2010. UK Data Service.

[ref33] R Core Team (2017) R: A Language and Environment for Statistical Computing.

[ref34] Slade M (2010) Mental illness and well-being: the central importance of positive psychology and recovery approaches. BMC Health Services Research 10, 26. 20102609 10.1186/1472-6963-10-26 PMC2835700

[ref35] Smith KE, Crosby RD, Wonderlich SA, Forbush KT, Mason TB and Moessner M (2018) Network analysis: an innovative framework for understanding eating disorder psychopathology. International Journal of Eating Disorders 51, 214–222. 29451959 10.1002/eat.22836 PMC5946321

[ref36] St Clair MC, Neufeld S, Jones PB, Fonagy P, Bullmore ET, Dolan RJ, Moutoussis M, Toseeb U and Goodyer IM (2017) Characterising the latent structure and organisation of self-reported thoughts, feelings and behaviours in adolescents and young adults. PLoS ONE 12, e0175381. 28403164 10.1371/journal.pone.0175381 PMC5389661

[ref37] Stochl J, Khandaker GM, Lewis G, Perez J, Goodyer IM, Zammit S, Sullivan S, Croudace TJ and Jones PB (2015) Mood, anxiety and psychotic phenomena measure a common psychopathological factor. Psychological Medicine 45, 1483–1493. 25394403 10.1017/S003329171400261X

[ref38] Tennant R, Hiller L, Fishwick R, Platt S, Joseph S, Weich S, Parkinson J, Secker J and Stewart-Brown S (2007) The Warwick-Edinburgh Mental Well-being Scale (WEMWBS): development and UK validation. Health and Quality of Life Outcomes 5, 63–63. 18042300 10.1186/1477-7525-5-63 PMC2222612

[ref39] University of London (2012) National Child Development Study: Sweep 8, 2008–2009. UK Data Service.

[ref40] van Borkulo C, Boschloo L, Borsboom D, Penninx BWJH, Waldorp LJ and Schoevers RA (2015) Association of symptom network structure with the course of depression. JAMA Psychiatry 72, 1219–1226. 26561400 10.1001/jamapsychiatry.2015.2079

[ref41] van Borkulo CD, with contributions from Sacha E and Millner A (2016) NetworkComparisonTest: Statistical Comparison of Two Networks Based on Three Invariance Measures.

[ref42] Weare K and Nind M (2011) Mental health promotion and problem prevention in schools: what does the evidence say? Health Promotion International 26, i29–i69. 22079935 10.1093/heapro/dar075

[ref43] World Health Organization (2002) Prevention and promotion in mental health.

[ref44] World Health Organization (2004) Promoting mental health: concepts, emerging evidence, practice: summary report.

