# Supplementary material for: Identifying key targets for interventions to improve psychological wellbeing: replicable results from four UK cohorts
Source: Psychol Med. 2018 Nov 15;49(14):2389–96. doi: 10.1017/S0033291718003288 (PMC6763534; doi:10.1017/S0033291718003288)
Supplement: Supplementary file 1 [file S0033291718003288sup.zip › S0033291718003288sup001.docx]

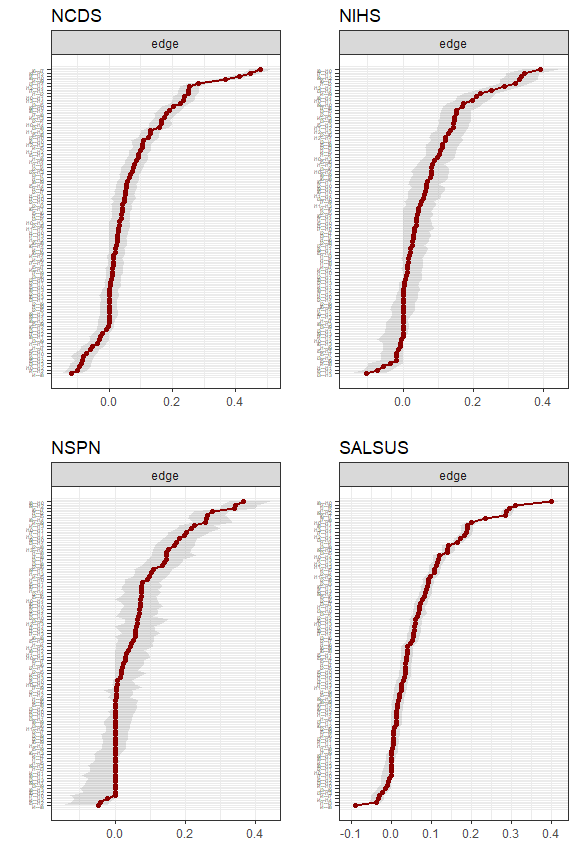

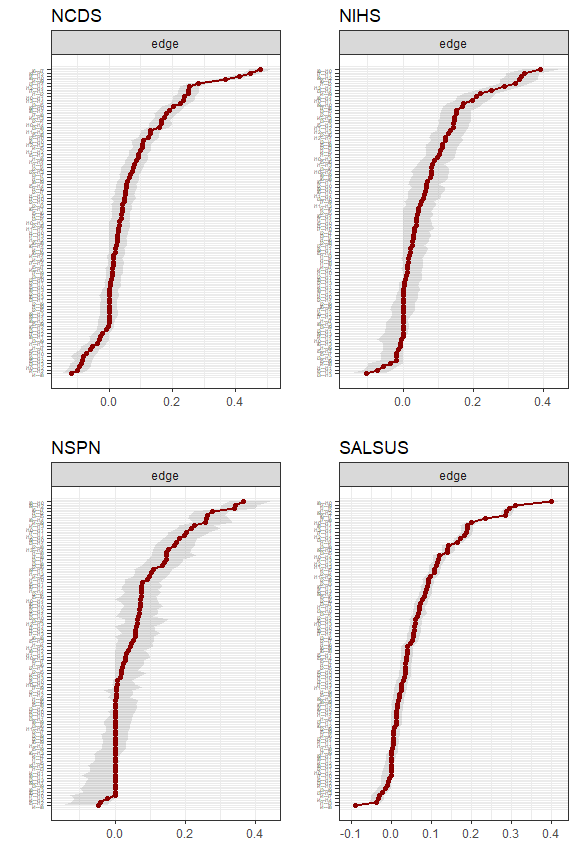


**Supplemental Figure 1: Point estimates (red) and 95% bootstrap confidence intervals (grey) of network edges (representing partial correlations between items).**
