# Supplementary material for: Identifying key targets for interventions to improve psychological wellbeing: replicable results from four UK cohorts
Source: Psychol Med. 2018 Nov 15;49(14):2389–96. doi: 10.1017/S0033291718003288 (PMC6763534; doi:10.1017/S0033291718003288)
Supplement: Supplementary file 1 [file S0033291718003288sup.zip › S0033291718003288sup003.docx]

####################################### R code used for analyses ########################################

# The following code shows the script for our analysis. We had data from four cohorts stored in R as four separate data.frames objects called wemwbs_ncds, wemwbs_nihs, wemwbs_nspn and wemwbs_sals. The structure of each data.frame is outlined below and was the same across all cohorts.

# head(wemwbs_ncds)

| # | ID | sex | i1 | i2 | i3 | i4 | i5 | i6 | i7 | i8 | i9 | i10 | i11 | i12 | i13 | i14 |
| --- | --- | --- | --- | --- | --- | --- | --- | --- | --- | --- | --- | --- | --- | --- | --- | --- |
| # | N10001N | Female | 4 | 3 | 5 | 5 | 4 | 5 | 5 | 5 | 4 | 5 | 5 | 2 | 5 | 4 |
| # | N10002P | Male | 4 | 4 | 4 | 2 | 4 | 4 | 4 | 4 | 3 | 4 | 5 | 5 | 4 | 4 |
| # | N10007U | Female | 4 | 4 | 4 | 4 | 4 | 5 | 5 | 5 | 5 | 5 | 5 | 5 | 5 | 5 |
| # | N10008V | Male | 4 | 4 | 4 | 5 | 5 | 5 | 5 | 5 | 4 | 3 | 5 | 5 | 5 | 4 |
| # | N10009W | Male | 4 | 4 | 4 | 4 | 3 | 4 | 4 | 4 | 3 | 4 | 5 | 4 | 4 | 4 |
| # | N10011Q | Male | 4 | 4 | 3 | 4 | 3 | 4 | 4 | 4 | 4 | 4 | 4 | 4 | 4 | 4 |

# sets working directory

setwd("D:/..../") # please insert the path to working directory where you want to save figures

# installs required packages

install.packages(c("qgpraph", "NetworkComparisonTest", "bootnet","networktools", "ggplot2", "gridExtra","EstimateGroupNetwork", "mgm", "reshape", "lemon", "dplyr"), dependencies=T)

# loads required packages

require(qgraph); require(NetworkComparisonTest); require(bootnet); require(networktools); require(ggplot2); require(gridExtra); require(EstimateGroupNetwork); require(mgm); require(reshape); require(lemon); require(dplyr)

# excludes individuals with missing data

wemwbs_ncds <- na.omit(wemwbs_ncds)

wemwbs_nihs <- na.omit(wemwbs_nihs)

wemwbs_nspn <- na.omit(wemwbs_nspn)

wemwbs_sals <- na.omit(wemwbs_sals)

# makes Table 1: WEMWBS item labels, wording, and item means (standard deviations) across samples

temp1 <- round(colMeans(wemwbs_ncds[,3:16], na.rm = TRUE),2)

temp2 <- round(colMeans(wemwbs_nihs[,3:16], na.rm = TRUE),2)

temp3 <- round(colMeans(wemwbs_nspn[,3:16], na.rm = TRUE),2)

temp4 <- round(colMeans(wemwbs_sals[,3:16], na.rm = TRUE),2)

temp1 <- paste(temp1, " (", round(apply(wemwbs_ncds[,3:16], 2, sd),2), ")", sep="")

temp2 <- paste(temp2, " (", round(apply(wemwbs_nihs[,3:16], 2, sd),2), ")", sep="")

temp3 <- paste(temp3, " (", round(apply(wemwbs_nspn[,3:16], 2, sd),2), ")", sep="")

temp4 <- paste(temp4, " (", round(apply(wemwbs_sals[,3:16], 2, sd),2), ")", sep="")

itemstats <- data.frame('Item label'= paste("i",1:14, sep=""), Statement=c(

"I have been feeling optimistic about the future",

"I have been feeling useful",

"I have been feeling relaxed",

"I have been feeling interested in other people",

"I have had energy to spare",

"I have been dealing with problems well",

"I have been thinking clearly",

"I have been feeling good about myself",

"I have been feeling close to other people",

"I have been feeling confident",

"I have been able to make up my own mind about things",

"I have been feeling loved",

"I have been interested in new things",

"I have been feeling cheerful"), 'NCDS'=temp1, 'NIHS'=temp2, 'NSPN'=temp3, 'SALSUS'=temp4)

itemstats

# estimates networks using mgm package and computes node predictability

temp1 <- mgm(wemwbs_ncds[,3:16], type=rep('g', 14), lev=rep(1,14), k=2)

pred_ncds <- predict(temp1, wemwbs_ncds[,3:16], error.continuous='VarExpl')

temp2 <- mgm(wemwbs_nihs[,3:16], type=rep('g', 14), lev=rep(1,14), k=2)

pred_nihs <- predict(temp2, wemwbs_nihs[,3:16], error.continuous='VarExpl')

temp3 <- mgm(wemwbs_nspn[,3:16], type=rep('g', 14), lev=rep(1,14), k=2)

pred_nspn <- predict(temp3, wemwbs_nspn[,3:16], error.continuous='VarExpl')

temp4 <- mgm(wemwbs_sals[,3:16], type=rep('g', 14), lev=rep(1,14), k=2)

pred_sals <- predict(temp4, wemwbs_sals[,3:16], error.continuous='VarExpl')

# computes fused graphical LASSO networks

groupnetwork_kfold <- EstimateGroupNetwork(list(wemwbs_ncds[,3:16],wemwbs_nihs[,3:16],wemwbs_nspn[,3:16],wemwbs_sals[,3:16]),inputType = "list.of.dataframes", covfun = cor_auto, method = "crossvalidation", strategy = "sequential", k = 10, seed=1234, criterion = c("ebic", "bic", "aic"), count.unique = FALSE, optimize = TRUE, optmethod = "CG", penalty = "fused", weights = "equal", penalize.diagonal = FALSE, ncores = 4, simplifyOutput = FALSE)

# makes Figure 1: Networks of WEMWBS items in four general population samples

png("Figure 1.png", width=10, height=10, units = "in", res = 600)

par(mfrow=c(2,2))

g1 <- qgraph(groupnetwork_kfold$network[[1]], layout = "spring",theme="colorblind", pie=pred_ncds$errors$Error.R2, border.width=2, vsize=10, border.color='#555555', label.color="#555555", color="#EEEEEE",DoNotPlot=TRUE); plot(g1); title("NCDS",adj=0, font.main=1, line=2.5)

g2 <- qgraph(groupnetwork_kfold$network[[2]], layout = "spring", theme="colorblind", pie=pred_nihs$errors$Error.R2, border.width=2, vsize=10, border.color='#555555', label.color="#555555", color="#EEEEEE",DoNotPlot=TRUE); plot(g2); title("NIHS",adj=0, font.main=1, line=2.5)

g3 <- qgraph(groupnetwork_kfold$network[[3]], layout = "spring", theme="colorblind", pie=pred_nspn$errors$Error.R2, border.width=2, vsize=10, border.color='#555555', label.color="#555555", color="#EEEEEE",DoNotPlot=TRUE); plot(g3); title("NSPN",adj=0, font.main=1, line=2.5)

g4 <- qgraph(groupnetwork_kfold$network[[4]], layout = "spring", theme="colorblind", pie=pred_sals$errors$Error.R2, border.width=2, vsize=10, border.color='#555555', label.color="#555555", color="#EEEEEE",DoNotPlot=TRUE); plot(g4); title("SALSUS",adj=0, font.main=1, line=2.5)

dev.off()

# assesses network differences across cohorts

comp_ncds_nihs <- NCT(wemwbs_ncds[,3:16], wemwbs_nihs[,3:16], it=5000, binary.data=FALSE, paired=FALSE, weighted=TRUE, test.edges=TRUE, progressbar=TRUE, edges='all')

comp_ncds_nspn <- NCT(wemwbs_ncds[,3:16], wemwbs_nspn[,3:16], it=5000, binary.data=FALSE, paired=FALSE, weighted=TRUE, test.edges=TRUE, progressbar=TRUE, edges='all')

comp_ncds_sals <- NCT(wemwbs_ncds[,3:16], wemwbs_sals[,3:16], it=5000, binary.data=FALSE, paired=FALSE, weighted=TRUE, test.edges=TRUE, progressbar=TRUE, edges='all')

comp_nihs_nspn <- NCT(wemwbs_nihs[,3:16], wemwbs_nspn[,3:16], it=5000, binary.data=FALSE, paired=FALSE, weighted=TRUE, test.edges=TRUE, progressbar=TRUE, edges='all')

comp_nihs_sals <- NCT(wemwbs_nihs[,3:16], wemwbs_sals[,3:16], it=5000, binary.data=FALSE, paired=FALSE, weighted=TRUE, test.edges=TRUE, progressbar=TRUE, edges='all')

comp_nspn_sals <- NCT(wemwbs_nspn[,3:16], wemwbs_sals[,3:16], it=5000, binary.data=FALSE, paired=FALSE, weighted=TRUE, test.edges=TRUE, progressbar=TRUE, edges='all')

comp_ncds_nihs$glstrinv.sep # shows network strength

comp_ncds_nihs$glstrinv.pval # p-value for global strength difference

comp_ncds_nihs$einv.pvals[comp_ncds_nihs$einv.pvals$`p-value`<0.05,] #shows which edges are statistically significant

comp_ncds_nspn$glstrinv.sep

comp_ncds_nspn$glstrinv.pval

comp_ncds_nspn$einv.pvals[comp_ncds_nspn$einv.pvals$`p-value`<0.05,]

comp_ncds_sals$glstrinv.sep

comp_ncds_sals$glstrinv.pval

comp_ncds_sals$einv.pvals[comp_ncds_sals$einv.pvals$`p-value`<0.05,]

comp_nihs_nspn$glstrinv.sep

comp_nihs_nspn$glstrinv.pval

comp_nihs_nspn$einv.pvals[comp_nihs_nspn$einv.pvals$`p-value`<0.05,]

comp_nihs_sals$glstrinv.sep

comp_nihs_sals$glstrinv.pval

comp_nihs_sals$einv.pvals[comp_nihs_sals$einv.pvals$`p-value`<0.05,]

comp_nspn_sals$glstrinv.sep

comp_nspn_sals$glstrinv.pval

comp_nspn_sals$einv.pvals[comp_nspn_sals$einv.pvals$`p-value`<0.05,]

# computes average layout

Layout <- averageLayout(groupnetwork_kfold$network[[1]],groupnetwork_kfold$network[[2]],groupnetwork_kfold$network[[3]],groupnetwork_kfold$network[[4]])

# makes Figure 2: Networks of WEMWBS items in four general population samples using average spring layout

png("Figure 2.png", width=10, height=10, units = "in", res = 600)

par(mfrow=c(2,2))

g1 <- qgraph(groupnetwork_kfold$network[[1]], layout = Layout, theme="colorblind", pie=pred_ncds$errors$Error.R2, border.width=2, vsize=10, border.color='#555555', label.color="#555555", color="#EEEEEE",DoNotPlot=TRUE); plot(g1); title("NCDS",adj=0, font.main=1, line=2.5)

g2 <- qgraph(groupnetwork_kfold$network[[2]], layout = Layout, theme="colorblind", pie=pred_nihs$errors$Error.R2, border.width=2, vsize=10, border.color='#555555', label.color="#555555", color="#EEEEEE",DoNotPlot=TRUE); plot(g2); title("NIHS",adj=0, font.main=1, line=2.5)

g3 <- qgraph(groupnetwork_kfold$network[[3]], layout = Layout, theme="colorblind", pie=pred_nspn$errors$Error.R2, border.width=2, vsize=10, border.color='#555555', label.color="#555555", color="#EEEEEE",DoNotPlot=TRUE); plot(g3); title("NSPN",adj=0, font.main=1, line=2.5)

g4 <- qgraph(groupnetwork_kfold$network[[4]], layout = Layout, theme="colorblind", pie=pred_sals$errors$Error.R2, border.width=2, vsize=10, border.color='#555555', label.color="#555555", color="#EEEEEE",DoNotPlot=TRUE); plot(g4); title("SALSUS",adj=0, font.main=1, line=2.5)

dev.off()

# bootstraps networks

set.seed("12345")

boot_networklasso_ncds <- bootnet(wemwbs_ncds[,3:16], nBoots = 2500, default = "EBICglasso", type = "nonparametric", nCores = 4, verbose = TRUE, computeCentrality =TRUE,lambda.min.ratio=0.001)

boot_networklasso_nihs <- bootnet(wemwbs_nihs[,3:16], nBoots = 2500, default = "EBICglasso", type = "nonparametric", nCores = 4, verbose = TRUE, computeCentrality =TRUE,lambda.min.ratio=0.001)

boot_networklasso_nspn <- bootnet(wemwbs_nspn[,3:16], nBoots = 2500, default = "EBICglasso", type = "nonparametric", nCores = 4, verbose = TRUE, computeCentrality =TRUE,lambda.min.ratio=0.001)

boot_networklasso_sals <- bootnet(wemwbs_sals[,3:16], nBoots = 2500, default = "EBICglasso", type = "nonparametric", nCores = 4, verbose = TRUE, computeCentrality =TRUE,lambda.min.ratio=0.001)

# makes Supplementary Figure 1: Point estimates (red) and 95% bootstrap confidence intervals (grey) of network edges (representing partial correlations between items)

png("Supplementary Figure 1.png", width=10, height=10, units = "in", res = 600)

p1 <- plot(boot_networklasso_ncds, statistics=c("edge"), plot="area", CIstyle="quantiles", order="sample", legend=FALSE) + ggtitle("NCDS") + theme(axis.text.y = element_text(size=4))

p2 <- plot(boot_networklasso_nihs, statistics=c("edge"), plot="area", CIstyle="quantiles", order="sample", legend=FALSE) + ggtitle("NIHS") + theme(axis.text.y = element_text(size=4))

p3 <- plot(boot_networklasso_nspn, statistics=c("edge"), plot="area", CIstyle="quantiles", order="sample", legend=FALSE) + ggtitle("NSPN") + theme(axis.text.y = element_text(size=4))

p4 <- plot(boot_networklasso_sals, statistics=c("edge"), plot="area", CIstyle="quantiles", order="sample", legend=FALSE) + ggtitle("SALSUS") + theme(axis.text.y = element_text(size=4))

grid.arrange(p1, p2, p3, p4, nrow =1)

dev.off()

# correlations presented in Supplementary Table 1: Spearman correlations between edges

cor(getWmat(g1)[lower.tri(getWmat(g1))], getWmat(g2)[lower.tri(getWmat(g2))], method="spearman") #0.87

cor(getWmat(g1)[lower.tri(getWmat(g1))], getWmat(g3)[lower.tri(getWmat(g3))], method="spearman") #0.79

cor(getWmat(g1)[lower.tri(getWmat(g1))], getWmat(g4)[lower.tri(getWmat(g4))], method="spearman") #0.80

cor(getWmat(g2)[lower.tri(getWmat(g2))], getWmat(g3)[lower.tri(getWmat(g3))], method="spearman") #0.75

cor(getWmat(g2)[lower.tri(getWmat(g2))], getWmat(g4)[lower.tri(getWmat(g4))], method="spearman") #0.82

cor(getWmat(g3)[lower.tri(getWmat(g3))], getWmat(g4)[lower.tri(getWmat(g4))], method="spearman") #0.83

mean(c(0.87,0.79,0.80,0.75,0.82,0.83)) #computes mean correlation (=0.81)

# makes Figure 3: Centrality indices across cohorts

strength <- as.data.frame(cbind(scale(centrality(g1)$InDegree), scale(centrality(g2)$InDegree), scale(centrality(g3)$InDegree), scale(centrality(g4)$InDegree)))

closeness <- as.data.frame(cbind(scale(centrality(g1)$Closeness), scale(centrality(g2)$Closeness), scale(centrality(g3)$Closeness), scale(centrality(g4)$Closeness)))

betweenness <- as.data.frame(cbind(scale(centrality(g1)$Betweenness), scale(centrality(g2)$Betweenness), scale(centrality(g3)$Betweenness), scale(centrality(g4)$Betweenness)))

strength <- mutate(strength, id = rownames(strength))

closeness <- mutate(closeness, id = rownames(closeness))

betweenness <- mutate(betweenness, id = rownames(betweenness))

colnames(strength)<-c("NCDS", "NIHS", "NSPN", "SALSUS", "Symptoms")

colnames(closeness)<-c("NCDS", "NIHS", "NSPN", "SALSUS", "Symptoms")

colnames(betweenness)<-c("NCDS", "NIHS", "NSPN", "SALSUS", "Symptoms")

strength_long <- melt(strength, id="Symptoms")

strength_long$Symptoms <- rep(1:14,4)

names(strength_long)[2] <- "Cohorts"

closeness_long <- melt(closeness, id="Symptoms")

closeness_long$Symptoms <- rep(1:14,4)

names(closeness_long)[2] <- "Cohorts"

betweenness_long <- melt(betweenness, id="Symptoms")

betweenness_long$Symptoms <- rep(1:14,4)

names(betweenness_long)[2] <- "Cohorts"

png("Figure 3.png", width=6, height=6, units = "in", res = 600)

p5 <- ggplot(data=strength_long, aes(x=Symptoms, y=value, colour=Cohorts)) + geom_line(size=1, aes(linetype=Cohorts)) + geom_point(shape = 21, fill = "white", size = 1.5, stroke = 1) + xlab(" ") + ylab("Centrality") + scale_y_continuous(limits = c(-3, 3)) + scale_x_continuous(breaks=c(1:14),labels=strength$Symptoms) + theme_bw() + theme(panel.grid.minor=element_blank(), axis.text.x = element_text(angle = 60, hjust = 1),legend.position="none") + ggtitle("Strength") + scale_linetype_manual(values=c("solid", "twodash", "dotted", "dashed"))

p6 <- ggplot(data=closeness_long, aes(x=Symptoms, y=value, colour=Cohorts)) + geom_line(size=1, aes(linetype=Cohorts)) + geom_point(shape = 21, fill = "white", size = 1.5, stroke = 1) + xlab(" ") + ylab("Centrality") + scale_y_continuous(limits = c(-3, 3)) + scale_x_continuous(breaks=c(1:14),labels=closeness$Symptoms) + theme_bw() + theme(panel.grid.minor=element_blank(), axis.text.x = element_text(angle = 60, hjust = 1),legend.position="none") + ggtitle("Closeness") + scale_linetype_manual(values=c("solid", "twodash", "dotted", "dashed"))

p7 <- ggplot(data=betweenness_long, aes(x=Symptoms, y=value, colour=Cohorts)) + geom_line(size=1, aes(linetype=Cohorts)) + geom_point(shape = 21, fill = "white", size = 1.5, stroke = 1) + xlab(" ") + ylab("Centrality") + scale_y_continuous(limits = c(-3, 3)) + scale_x_continuous(breaks=c(1:14),labels=betweenness$Symptoms) + theme_bw() + theme(panel.grid.minor=element_blank(), axis.text.x = element_text(angle = 60, hjust = 1),legend.position="none") + ggtitle("Betweenness") + scale_linetype_manual(values=c("solid", "twodash", "dotted", "dashed"))

p7 <- grid_arrange_shared_legend(p7, position='bottom', plot=FALSE)

grid.arrange(p5, p6, p7, nrow =3)

dev.off()

# case dropping bootstrap

set.seed("12345")

boot_networklasso_centrality_ncds <- bootnet(wemwbs_ncds[,3:16], nBoots = 2500, default = "EBICglasso", type = "case", nCores = 4, statistics = c("strength","closeness","betweenness"), model = "GGM", verbose = TRUE, computeCentrality = TRUE,lambda.min.ratio=0.001)

boot_networklasso_centrality_nihs <- bootnet(wemwbs_nihs[,3:16], nBoots = 2500, default = "EBICglasso", type = "case", nCores = 4, statistics = c("strength","closeness","betweenness"), model = "GGM", verbose = TRUE, computeCentrality = TRUE,lambda.min.ratio=0.001)

boot_networklasso_centrality_nspn <- bootnet(wemwbs_nspn[,3:16], nBoots = 2500, default = "EBICglasso", type = "case", nCores = 4, statistics = c("strength","closeness","betweenness"), model = "GGM", verbose = TRUE, computeCentrality = TRUE,lambda.min.ratio=0.001)

boot_networklasso_centrality_sals <- bootnet(wemwbs_sals[,3:16], nBoots = 2500, default = "EBICglasso", type = "case", nCores = 4, statistics = c("strength","closeness","betweenness"), model = "GGM", verbose = TRUE, computeCentrality = TRUE,lambda.min.ratio=0.001)

# makes Supplementary Figure 2: Stability of centrality indices: point estimates and corresponding 95% CIs

g_legend<-function(a.gplot){

tmp <- ggplot_gtable(ggplot_build(a.gplot))

leg <- which(sapply(tmp$grobs, function(x) x$name) == "guide-box")

legend <- tmp$grobs[[leg]]

return(legend)}

p1forlegend <- plot(boot_networklasso_centrality_ncds) + ylab("Correlation")

mylegend<-g_legend(p1forlegend)

png("Supplementary Figure 2.png", width=7, height=7, units = "in", res = 600)

p1 <- plot(boot_networklasso_centrality_ncds) + ylab("Correlation") + theme(legend.position="none") + ggtitle("NCDS") + scale_y_continuous(limits = c(0, 1))

p2 <- plot(boot_networklasso_centrality_nihs) + ylab("Correlation") + theme(legend.position="none") + ggtitle("NIHS") + scale_y_continuous(limits = c(0, 1))

p3 <- plot(boot_networklasso_centrality_nspn) + ylab("Correlation") + theme(legend.position="none") + ggtitle("NSPN") + scale_y_continuous(limits = c(0, 1))

p4 <- plot(boot_networklasso_centrality_sals) + ylab("Correlation") + theme(legend.position="none") + ggtitle("SALSUS") + scale_y_continuous(limits = c(0, 1))

grid.arrange(p1, p2, p3, p4, mylegend, nrow =3)

dev.off()

# computes cs coefficients

CS_ncds <- corStability(boot_networklasso_centrality_ncds)

CS_nihs <- corStability(boot_networklasso_centrality_nihs)

CS_nspn <- corStability(boot_networklasso_centrality_nspn)

CS_sals <- corStability(boot_networklasso_centrality_sals)

# computes values in Table 2: Correlation stability coefficients

CSfinal <- rbind(CS_ncds,CS_nihs,CS_nspn,CS_sals)

rownames(CSfinal) <- c("NCDS","NIHS", "NSPN", "SALSUS")

CSfinal

# assesses gender differences

gendercomparison_ncds <- NCT(wemwbs_ncds[wemwbs_ncds$sex=="Male",3:16], wemwbs_ncds[wemwbs_ncds$sex=="Female",3:16], it=5000, binary.data=FALSE, paired=FALSE, weighted=TRUE, test.edges=TRUE, edges='all', progressbar=TRUE)

gendercomparison_ncds$glstrinv.pval # p-value for global strength difference

gendercomparison_ncds$einv.pvals$Var1[gendercomparison_ncds$einv.pvals$`p-value`<0.05] #which items are involved in significant differences

gendercomparison_ncds$einv.pvals$Var2[gendercomparison_ncds$einv.pvals$`p-value`<0.05] #which items are involved in significant differences

gendercomparison_ncds$einv.pvals$`p-value`[gendercomparison_ncds$einv.pvals$`p-value`<0.05] #p-value

gendercomparison_nihs <- NCT(wemwbs_nihs[wemwbs_nihs$sex=="Male",3:16], wemwbs_nihs[wemwbs_nihs$sex=="Female",3:16], it=5000, binary.data=FALSE, paired=FALSE, weighted=TRUE, test.edges=TRUE, edges='all', progressbar=TRUE)

gendercomparison_nihs$glstrinv.pval

gendercomparison_nihs$einv.pvals$Var1[gendercomparison_nihs$einv.pvals$`p-value`<0.05]

gendercomparison_nihs$einv.pvals$Var2[gendercomparison_nihs$einv.pvals$`p-value`<0.05]

gendercomparison_nihs$einv.pvals$`p-value`[gendercomparison_nihs$einv.pvals$`p-value`<0.05]

gendercomparison_nspn <- NCT(wemwbs_nspn[wemwbs_nspn$sex=="Male",3:16], wemwbs_nspn[wemwbs_nspn$sex=="Female",3:16], it=5000, binary.data=FALSE, paired=FALSE, weighted=TRUE, test.edges=TRUE, edges='all', progressbar=TRUE)

gendercomparison_nspn$glstrinv.pval

gendercomparison_nspn$einv.pvals$Var1[gendercomparison_nspn$einv.pvals$`p-value`<0.05]

gendercomparison_nspn$einv.pvals$Var2[gendercomparison_nspn$einv.pvals$`p-value`<0.05]

gendercomparison_nspn$einv.pvals$`p-value`[gendercomparison_nspn$einv.pvals$`p-value`<0.05]

gendercomparison_sals <- NCT(wemwbs_sals[wemwbs_sals$sex=="Male",3:16], wemwbs_sals[wemwbs_sals$sex=="Female",3:16], it=5000, binary.data=FALSE, paired=FALSE, weighted=TRUE, test.edges=TRUE, edges='all', progressbar=TRUE)

gendercomparison_sals$glstrinv.pval

gendercomparison_sals$einv.pvals$Var1[gendercomparison_sals$einv.pvals$`p-value`<0.05]

gendercomparison_sals$einv.pvals$Var2[gendercomparison_sals$einv.pvals$`p-value`<0.05]

gendercomparison_sals$einv.pvals$`p-value`[gendercomparison_sals$einv.pvals$`p-value`<0.05]
