# Supplementary material for: Identifying key targets for interventions to improve psychological wellbeing: replicable results from four UK cohorts
Source: Psychol Med. 2018 Nov 15;49(14):2389–96. doi: 10.1017/S0033291718003288 (PMC6763534; doi:10.1017/S0033291718003288)
Supplement: Supplementary file 1 [file S0033291718003288sup.zip › S0033291718003288sup004.docx]

**Supplemental Table 1: Spearman correlations between edges.**

|  | **NCDS** | **NIHS** | **NSPN** | **SALSUS** |
| --- | --- | --- | --- | --- |
| **NCDS** | 1 | - | - | - |
| **NIHS** | 0.87 | 1 | - | - |
| **NSPN** | 0.79 | 0.75 | 1 | - |
| **SALSUS** | 0.80 | 0.82 | 0.83 | 1 |
