# Supplementary figures and images for: Identifying key targets for interventions to improve psychological wellbeing: replicable results from four UK cohorts
Source: Psychol Med. 2018 Nov 15;49(14):2389–96. doi: 10.1017/S0033291718003288 (PMC6763534; doi:10.1017/S0033291718003288)

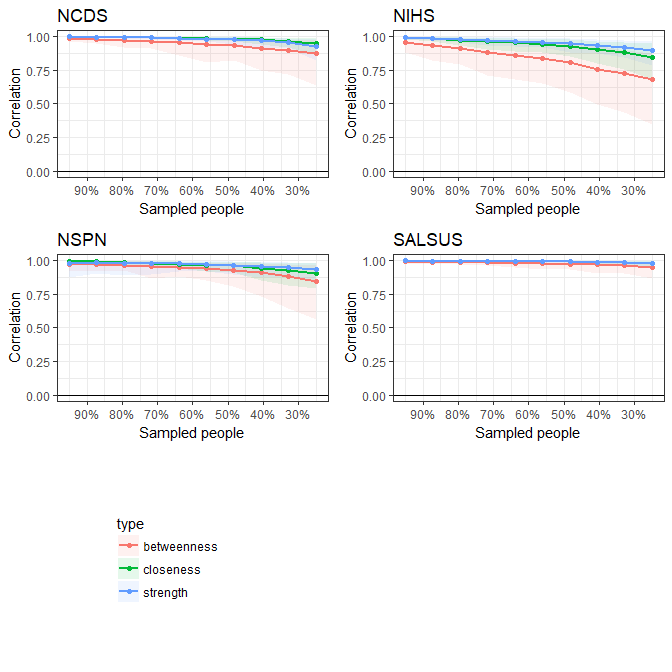

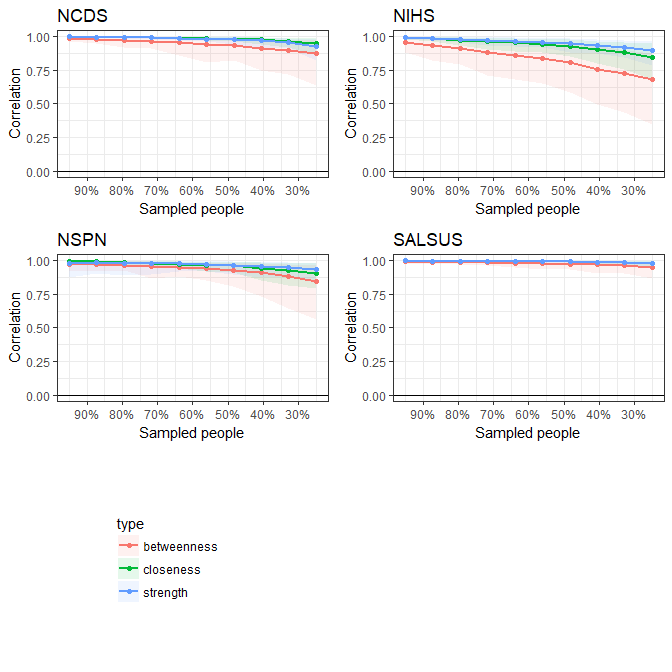


**Supplemental Figure 2: Stability of centrality indices: point estimates and corresponding 95% CIs.**

Supplement: Supplementary file 1 [file S0033291718003288sup.zip › S0033291718003288sup002.docx]
